# A dual-port THz Time Domain Spectroscopy System optimized for recovery of a sample’s Jones matrix

**DOI:** 10.1038/s41598-019-39322-y

**Published:** 2019-02-14

**Authors:** Guozhong Zhao, Giorgio Savini, Yang Yu, Shuai Li, Jin Zhang, Peter Ade

**Affiliations:** 1 Beijing Advanced Innovation Center for Imaging Theory and Technology, Capital Normal University, Beijing, China; 2 Department of Physics, Capital Normal University, Beijing, China; 3 Physics and Astronomy Department, University College London, London, WC1E 6BT UK; 4 School of Computing and Information Science, Anglia Ruskin University, Cambridge, CB1 1PT UK; 5 School of Physics and Astronomy, Cardiff University, Cardiff, CF24 3AA Wales UK

## Abstract

We describe the design, build and characterization of a novel two-output port configuration for a THz-Time Domain Spectroscopy (TDS) system. By introducing a tilted THz ultra-broadband polarizer, we split the THz beam in two orthogonal polarization detector branches. The probe laser is similarly split (with an optical polarizer) replicating the detection chain to obtain two independent orthogonal polarization detection units. We describe the system’s performance highlighting some of the advantages of this system in one of its two modes of operation: optimized polarimetry for Jones matrix measurements. A bi-refringent sapphire standard was measured to confirm its capabilities and assess the performance of the system showing good agreement with existing literature data.

## Introduction

With the rapid development of THz radiation generation and detection technology, THz time-domain spectroscopy (THz-TDS) has rapidly matured over the last 30 years^1–4^ and has become of mainstream use in research (medical and material science) as well as in industrial applications. At the same time, this has pushed the development of THz emitters^5–9^ and detectors^10–15^, improving systems significantly due to advances in device fabrication. Polarization sensitive THz spectroscopy has attracted the interest of researchers due to the importance of the anisotropic dielectric properties of materials in chemical, physical and biochemical fields^16–19^. Advances in THz photonics and metamaterials through the development of metal and metal-plus-dielectric based filters^20–23^, frequency selective surfaces (FSS)^24^, magnetic mirrors and artificial dielectric based coatings and lenses rely on good quality polarization spectroscopic measurements. It is through precise polarisation measurements that the modelling-measuring feedback allows manufacturing of the latter examples to improve in quality and repeatability. The applications of the latter devices span communications (vortex beam and orbital angular momentum)^25,26^, industrial non-invasive quality assurance^27–30^, as well as the more standard and well established bi-refringent crystal measurements and optically active devices.

In this paper, we show how a THz TDS system can be modified with the use of an ultra-broadband THz polarizer^31^ combined with the duplication of the detection chain of a standard THz TDS system to optimize it for use in polarimetry studies. The polarizer, placed downstream from the sample, splits the THz beam in two orthogonally polarized beams each detected with its independent detector (and probe beam). The synchronous detection of orthogonal polarization modes allows, with one single sample rotation, a clean extraction of Jones matrix elements of the sample of interest. In our system we additionally sought to assemble the system so to provide close similarity in both branches although it will be argued later that this is not a necessary requirement.

The beam where the sample is positioned is collimated to minimize optical coupling issues caused by the sample as well as reducing potential Gouy phase shift errors.

An additional application of this system is available in measurements where full information polarimetry is not required. In this case the sample to be measured can be placed downstream from the THz polarizing beam-splitter, allowing one to perform simultaneous sample and reference measurements in one single stage scan. This procedure avoids many of the uncertainties introduced by the variation of temperature, humidity and laser source power across the duration of scans. This second mode of operation is not explored in this paper.

## Results

### Experimental Setup

**Figure 1:**
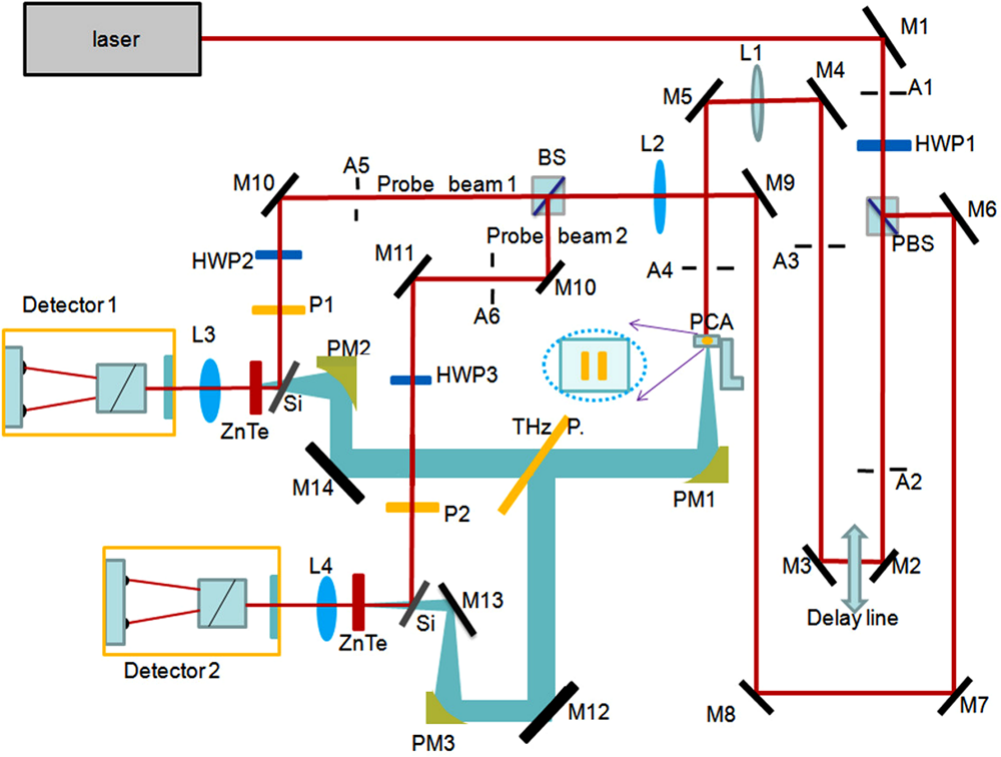
The overall system layout is shown with flat mirrors (M), optical lenses (L), off-axis parabolic mirrors for the THz beam (PM), iris apertures (A), half-wave plates (HWP) and polarizers (P). The THz polarizer is indicated with “THz P”. The PCA orientation is also shown providing horizontally polarized radiation. The two Silicon plates (Si) are also shown for THz/laser overlap and ZnTe crystals with the two detector units (with QWP + Wollaston prism) on the left.

A diagram of the entire system is shown in Fig. 1 where the similarity with the replication of the probe beam section post-optical delay is apparent. The system mainly includes: a mode-locked Ti:sapphire oscillators supplying 100 fs pulses with a repetition rate of 82 MHz and average power 3400 mW and the center wavelength of 800 nm. The laser is shared with other TDS systems thus providing an average power of 1300 mW to the pump beam for this dual-port system incident on a “home” grown gallium arsenide photoconductive antenna (PCA) to radiate THz power.

The THz beam is then collimated with the use of a gold-coated off-axis parabolic mirror (PM, placed as in Fig. 1) and an ultra-broadband photo-lithographic THz polarizer^31,32^ is used to separate the beam in two clean orthogonal polarization states (both collimated). The two beams are then refocused each with a similar PM on separate ZnTe crystals for optical rectification. Standard TDS THz detection systems comprised of a quarter-wave plate (QWP) and a Wollaston prism for differential detection with a balanced photo-detector pair are used in each port. Each probe beam, delayed with a linear optical path delay stage (Newport - ESP301), is similarly optically split with a polarizing optical beam. The differential output signals are amplified by a lock-in amplifier (LIA) referenced to the PCA voltage bias and sent to a computer for processing.

### The THz Polarizer

For ideal operation, the THz polarizer should be highly efficient in both transmission and reflection. In addition, to minimize port differences, an ultra-thin substrate for the polarizer is preferable. The first polarizer used was a photo-lithographic copper-based metal mesh with 10 *μm* periodic strips provided by Cardiff University on a 1.9 *μm* Mylar substrate which made its transmission port “echo” (the time domain standing wave signature) almost negligible within the main pulse.

**Figure 2:**
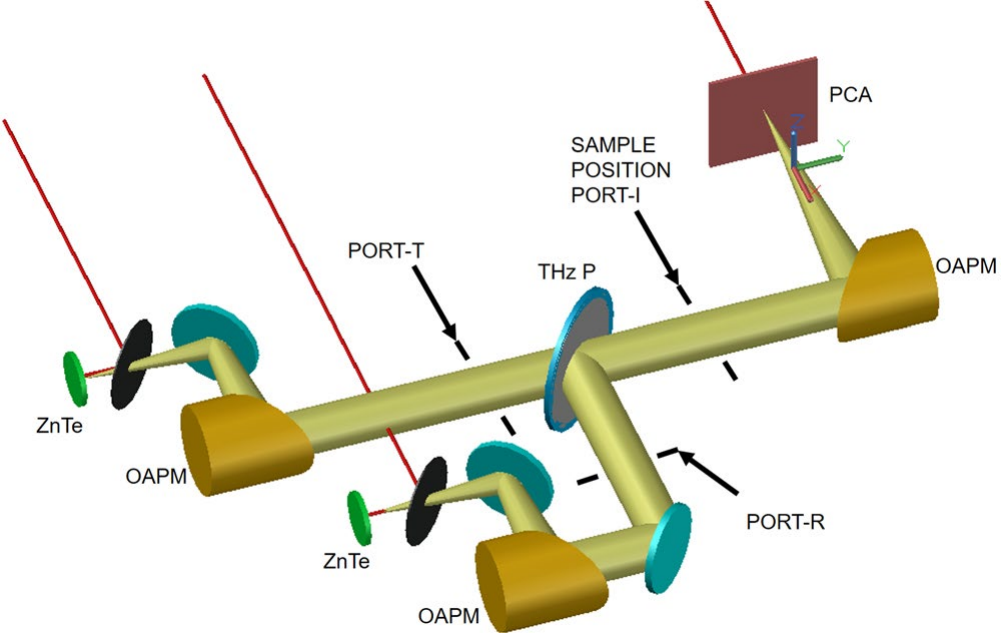
Detail of the THz section. The polarized beam splitter (PBS) is positioned in order to provide a projected 45 degree axis to the incoming polarization.

The polarizer is mounted at 45 degrees to split the beam in two perpendicular ports. Its axis is rotated to form a projected angle of 45 degrees as seen from the incident (I) port (Fig. 2). With the PCA antenna poles aligned perpendicularly to the optical bench, the THz signal generated is split by the polarizer in roughly equal parts. This allows to record background references on both ports thus removing optical efficiencies from sample transmission measurements (as is the case in standard single port systems).

An alternative option for the setup could be to orient the input polarization originating at the PCA at 45 degrees. This would facilitate the positioning of the polarizer by allowing a placement at 0 or 90 degrees which is less complex. Note that 90 degrees (i.e. a vertical E field) would imply that the polarizer grids would be aligned horizontally, thus not altering line density seen by the incoming wave on the polariser projection. However, we preferred a horizontal E field to avoid small additional polarization caused by the first off-axis mirror (hence our initial choice of PCA alignment).

**Figure 3:**
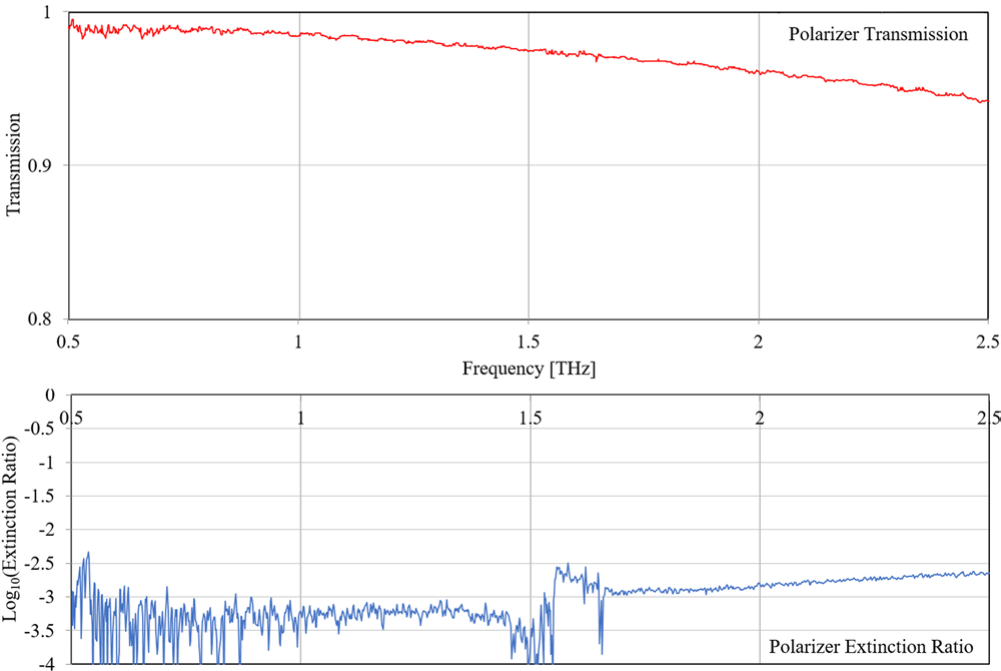
Top: Spectral Transmission of an identical photo-lithographic broadband polarizer to the one initially used in the system. Bottom: Extinction ratio of the same polarizer for the orthogonal polarization. This shows a transmission higher than 95% for frequencies above 2 THz and an overall rejection of the orthogonal mode lower than 1%.

A functional requirement on the polarizer is for its performance to be ultra-broadband allowing frequencies from as low as 100 GHz to over 2.5 THz to transmit and reflect efficiently. This polarizer was tested in a Fourier Transform Spectrometer and its measured on-axis transmission is shown in Fig. 3. An extinction curve of the same polarizer for the transmission of the orthogonal field is shown on the lower part of the same figure.

Following unfortunate damage of this high performing polarizer a replacement polarizer was obtained^33^ based on a similar photo-lithographic architecture with Al on polyamide with a noticeable substrate thickness (35 *μm*) in comparison.

**Figure 4:**
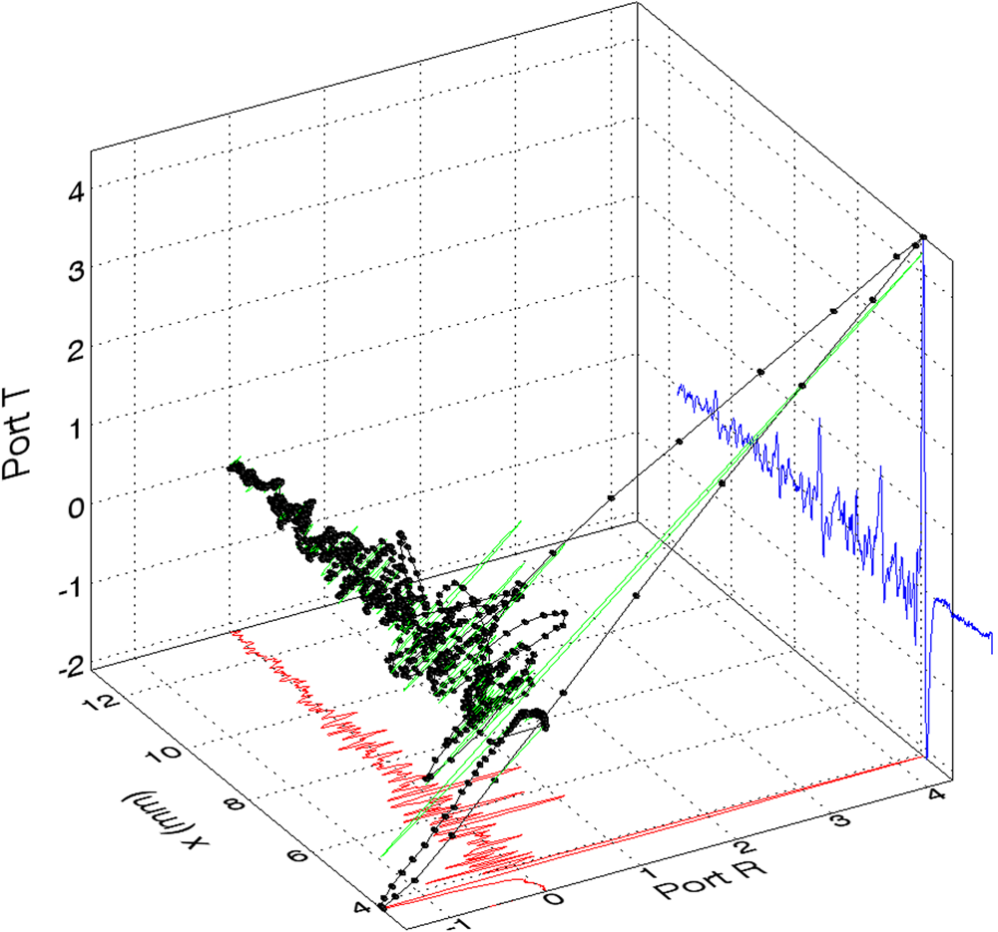
A 3D visualization of the two orthogonal simultaneously measured time-domain scans. The Red and Blue are the two port TDS scans where the Blue scan has been shifted in this representation to have coincident rise of the main THz pules. The green is the ideal case of two identical ports (created assuming two identical curves - like the red one - expecting thus a combination which lies in a plane close to that at 45 degrees). Black: The actual time-domain visualization of the combined signals.

Adopting this second polarizer, a small time-of-flight delay is apparent. This can be visualized by adopting a three-dimensional visualization of the two independent field responses and their time-domain combination in Fig. 4. The two time domain interferograms are projected on the “walls” of the box with the y-axis representing the time delay and the x- (red plot on the xy plane) and z-(blue plot on the yz plane) axes respectively the E field amplitude on ports T and R.

Due to the (non-negligible) thickness of the polarizer, the peak shapes and in particular the fall from the peak, will be slightly dissimilar and thus when combined show a loop-like structure. Aside from the visual representation (inspired by^19^), the analysis of the two independent detection ports is not affected in any way by this. The three echoes present in the two scans are traceable back to the PCA antenna substrate back-reflection, the silicon combiner used to reflect the probe laser beam and the final ZnTe crystal.

As per standard THz TDS operation, it is possible to remove/reduce these features through ratio of the Fourier Transforms with the reference set of data (that with the sample removed) or via reference template removal, which we decided to adopt.

We have hence performed a “complete” set of measurements of a bi-refringent sapphire standard (thickness of 1.5 mm) used as a HWP for a frequency of ≈0.3 THz held in a manual rotating mount with 0.5 degree accuracy and acquired scans at 5 degree intervals. The sapphire was placed in the incident beam with no particular axis alignment in order to avoid initial bias in the choice of measuring angles for the Jones matrix recovery.

For clarity, the ports are identified as “I” for the collimated beam incident on the polarizer, “T” and “R” for the beams transmitted through and reflected off the polarizer respectively.

### Jones Matrix recovery

The incident field generated at the PCA is horizontally aligned and parallel to the plane of the optical bench. Each of the probe beam polarizations is aligned to maximize the output signal on the relevant port. The latter step is done through amplitude optimization^34^ of the probe polarization and ZnTe axis orientation. This highlights the first advantage of this system with respect to a single port standard system: variation of polarization angle of the incident signal does not affect single port detection efficiency. This is the case when the THz signal is rotated changing the efficiency of the detection^34^ on a single port system.

The insertion of an element with a generic complex Jones matrix of which we wish to find the elements can then be written together with the Jones matrix for the polarizer at 45 degree in Transmission and Reflection respectively as:

1 $${D}_{{T}_{out}}=\frac{1}{4}\begin{array}{l}(1\,1)\end{array}\cdot (\begin{array}{cc}1 & 1\\ 1 & 1\end{array})\cdot (\begin{array}{cc}{j}_{11} & {j}_{12}\\ {j}_{21} & {j}_{22}\end{array})\cdot (\begin{array}{c}1\\ 0\end{array})$$

2 $${D}_{{R}_{out}}=\frac{1}{4}\begin{array}{l}(1\,-\,1)\end{array}\cdot (\begin{array}{cc}1 & -\,1\\ -\,1 & 1\end{array})\cdot (\begin{array}{cc}{j}_{11} & {j}_{12}\\ {j}_{21} & {j}_{22}\end{array})\cdot (\begin{array}{c}1\\ 0\end{array})$$

where on the right we have multiplied for the input field and on the left by the co-aligned detection. For the generic sample Jones matrix we then introduce rotation by an angle *θ* which we can then rewrite generically using the abbreviations $c=\,\cos \,\theta$ and $s=\,\sin \,\theta$ as

3 $${J}_{sample}(\theta )=(\begin{array}{cc}{c}^{2}{j}_{11}+{s}^{2}{j}_{22}-cs({j}_{12}+{j}_{21}) & cs({j}_{11}-{j}_{22})+{c}^{2}{j}_{12}-{s}^{2}{j}_{21}\\ cs({j}_{11}-{j}_{22})+{c}^{2}{j}_{21}-{s}^{2}{j}_{12} & {s}^{2}{j}_{11}+{c}^{2}{j}_{22}+cs({j}_{12}+{j}_{21})\end{array})$$

Substituting for the values of cosine and sine at 0 and 90 degrees we obtain the values for the four parameters:

4 $$\begin{array}{ll}{j}_{11}={D}_{T}(0^\circ )+{D}_{R}(0^\circ ) & {j}_{12}={D}_{R}(90^\circ )-{D}_{T}(90^\circ )\\ {j}_{21}={D}_{T}(0^\circ )-{D}_{R}(0^\circ ) & {j}_{22}={D}_{T}(90^\circ )+{D}_{R}(90^\circ )\end{array}$$

having omitted the double-subscript “out” used above for brevity. This specific representation and thus recovery of the parameters are for the values taken at 0 and 90 degrees in the “I” beam. Thus, a birefringent crystal would only return the expected diagonal matrix if placed knowingly with the axes aligned to the optical table reference frame.

A similar measurement can be performed with a standard TDS for the recovery of birefringent optical properties. However, this either relies on the prior knowledge of the axes position to measure the plate’s diagonal elements, or requires multiple angle rotations to determine the same. Furthermore, more complex devices that cause phase delay as well as some degree of absorption require both co-pol and cross-pol measurements at many angles for these to be disentangled.

### Birefringent sapphire measurements

The sapphire plate had a diameter of ≈9 cm (much wider than the entire collimated beam) and a thickness $d=1.495\pm 0.010\,{\rm{mm}}$. The latter error expresses the statistical values of measured thickness rather than measurement uncertainty. The plate was placed in the “I” beam on a rotating mount purposely without initial knowledge of angle between the crystal axes and the rotating mount reference (0°). Background reference scans were taken prior to the placement of the plate.

**Figure 5:**
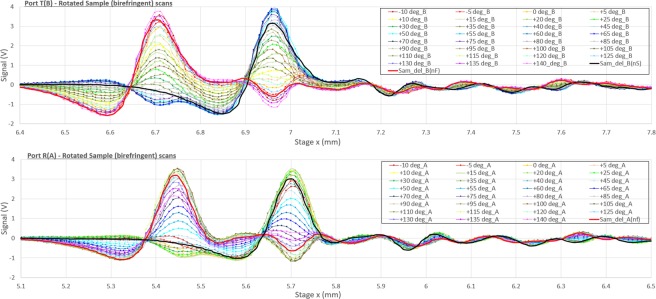
Close-up view of the sample main peak taken on the two ports as the sample is rotated in 5 degree steps. Top: the Transmission (T) port and bottom the Reflection (R) port. The color coding is such that scans taken simultaneously on the two ports for the same sample angle is the same. The thick black and red curves show the same port’s reference scan, scaled/shifted according to the sample’s internal reflection when the input field is aligned respectively to the ordinary and extraordinary axes (see text for numerical calculation).

Scans of the plate were taken with 5 degree steps of the rotating mount and recorded simultaneously on the two ports (T and R). Figure 5 shows the close-up view of measured time domain scans with the expected modulation taking place on the two ports (T-top and R-bottom) as the plate rotates. For comparison, the reference scans for each port were shifted by the time of flight relevant to the known thickness and either of the two sapphire refractive indices (extraordinary or ordinary) $d({n}_{e}-1)$ and $d({n}_{o}-1)$. The same scans are scaled in amplitude by a factor of $1-\frac{{(1-{n}_{e/o})}^{2}}{{(1+{n}_{e/o})}^{2}}$ which approximates well the reduction in amplitude from the single pass (for the respective amplitudes).

**Figure 6:**
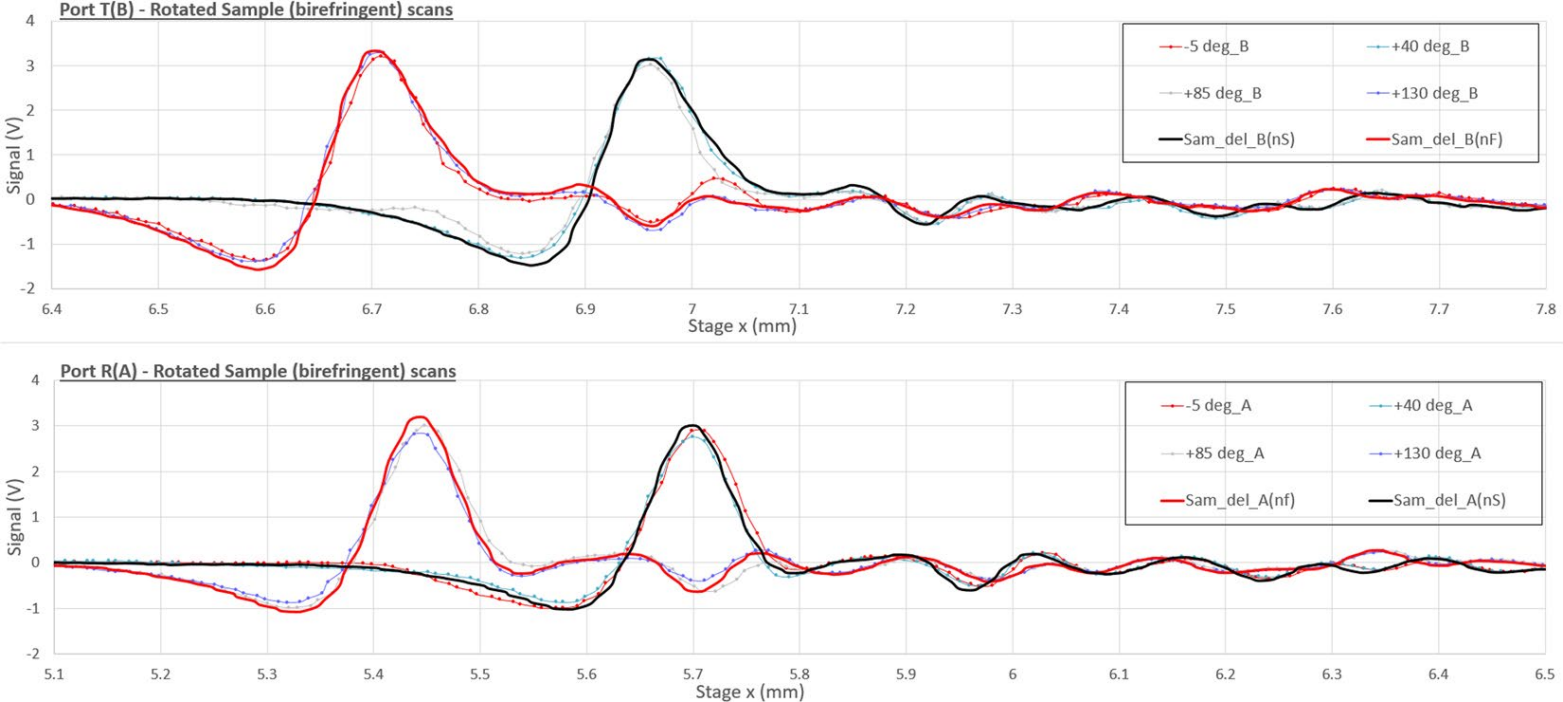
Similar close up to the above figure where the closest matching curves have been left showing the 45 degree periodicity expected by Jones manipulation.

To prove the Jones recovery we performed standard Fourier Transforms of the scans and show how these can be combined in pair sets to determine the complex Jones amplitudes as a function of frequency. We note, by removing all other curves in Fig. 5 and leaving the ones which closely match the shifted/scaled reference scans, we can identify the birefringent sample axes immediately as the 40/130 degree pair (Fig. 6). These two curves are the only pair of the closest four matching curves that present the same time of flight delay for both detectors. This is expected when the input field traverses the plate along a single crystal axis (with a precision better than ±2.5° given the angle sampling in the rotation of the plate).

These curves do not represent the two maximum achievable signal on the two detectors because of the 45 degree tilted polarizer which splits the signal on both ports. The maximum signal at each of the two peaks and on each port is a compromise between the closeness of the input field to the axis relevant to the peak position and its modulated output making it through to one or the other channel.

We consider now this subset of two scans taken with 90 degree difference in order to apply the algorithm above and extract the Jones matrix of the sample (which we can easily verify, knowing the properties of the birefringent crystal relatively accurately).

Common echo signatures (ZnTe, Si and PCA) are removed with subtracted reference templates from the time scans. These are then Fourier transformed and the ratio with reference scans calculated to yield transmission spectra for each measurement angle. These are identified in the expressions as *D*_*T*_ or *D*_*R*_ depending if they are detected from the Transmission or Reflection port and by the angle in parenthesis.

**Figure 7:**
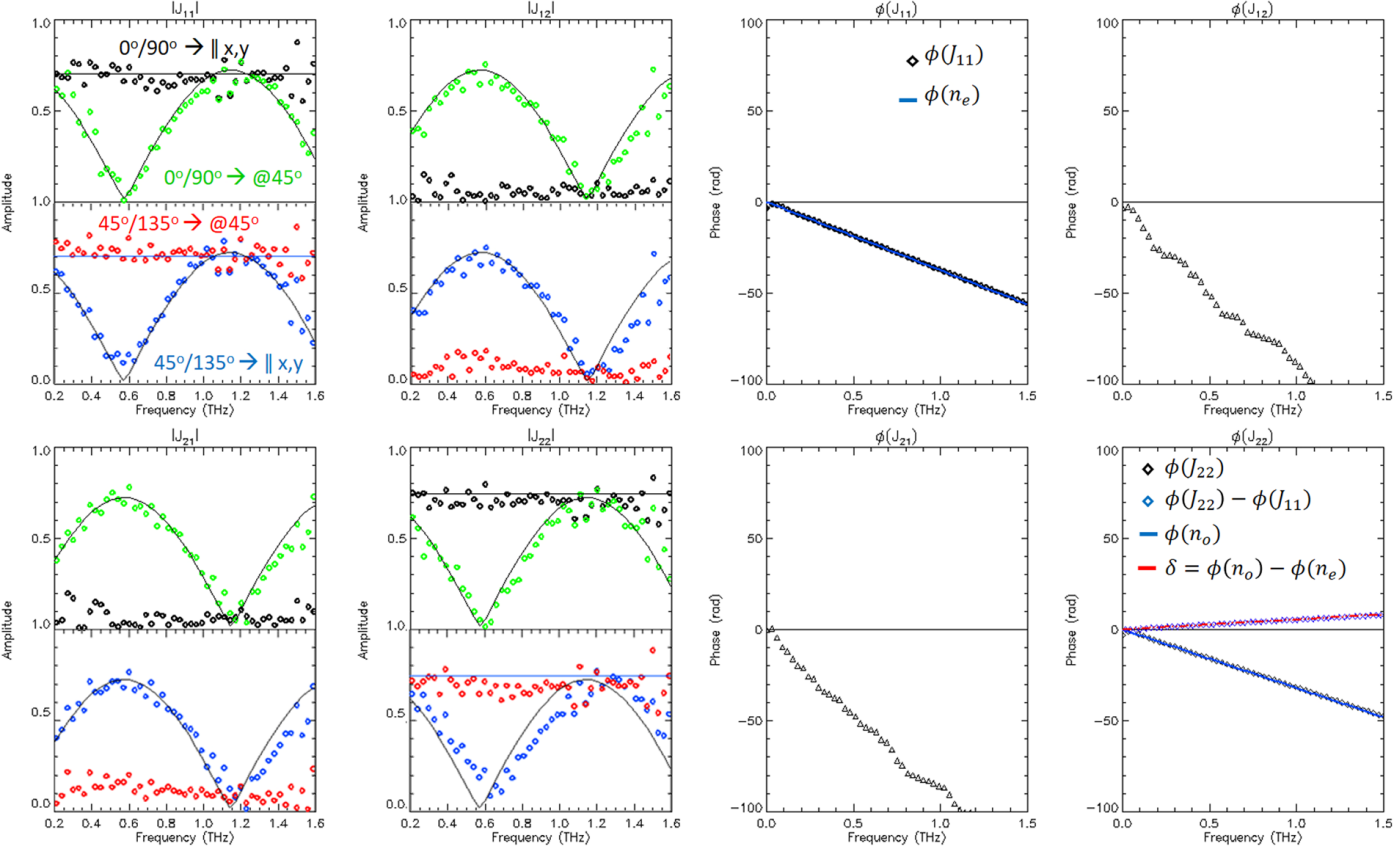
Jones matrix elements arranged with the amplitudes on the left and complex phase on the right. In the four left plots arranged as the Jones matrix itself, the black dots represent the spectral ratios of the samples recorded in the plate mount as 40° and 130° (which are the measurements with the closest alignment of sapphire axis to the XY reference). These are then combined as described in 4. The same is done for sample measurements recorded as −5° and 85° (blue dots) in the lower half of the same plots. See text for further explanation. The right block of four plots show the recovered phase from the same combination of ratios, with the matching expected linear phases when the axes align with those corresponding to the sample thickness of birefringent sapphire.

The combination of the aligned scans are shown as the black dot sets in Fig. 7. The eight plots describe the amplitude (left) and phase (right) of the four Jones matrix elements. The phases are only shown for the aligned scan sets as these are easy to compare and validate with existing sapphire data. The phase delay matches the data quite closely (after phase unwrapping).

In our experiment, the use of a tilted bi-refringent crystal as a sample has also the purpose of “simulating” a sample which alters the polarisation content of the x and y axis (which would require detector tuning in order to recover its generic Jones matrix). The results of the analysis and for that of two scans rotated at 45 degrees with respect to the first two are plotted alongside as the blue dots.

For verification we compared this output with the actual Jones matrix of a birefringent plate in a reference frame tilted by 45 degree (overplotted line).

Further verification was performed by calculating the analogous to the above equations for another non-zero angle (for which we will use 45 degrees, but any other angle could be used) and assume that this is the device’s reference frame. Thus

5 $$\begin{array}{rcl}{J}_{device}(0^\circ ) & = & {J}_{sample}(45^\circ )\\  & = & (\begin{array}{cc}{d}_{11} & {d}_{12}\\ {d}_{21} & {d}_{22}\end{array})\\  & = & \frac{1}{2}(\begin{array}{cc}({j}_{11}+{j}_{22})-({j}_{12}+{j}_{21}) & ({j}_{11}-{j}_{22})+({j}_{12}-{j}_{21})\\ ({j}_{11}-{j}_{22})-({j}_{12}-{j}_{21}) & ({j}_{11}+{j}_{22})+({j}_{12}+{j}_{21})\end{array})\end{array}$$

6 $$\begin{array}{rcl}{J}_{device}(90^\circ ) & = & {J}_{sample}(135^\circ )\\  & = & (\begin{array}{cc}{d}_{22} & \,-{d}_{21}\\ \,-{d}_{12} & {d}_{11}\end{array})\\  & = & \frac{1}{2}(\begin{array}{cc}({j}_{11}+{j}_{22})+({j}_{12}+{j}_{21}) & \,-({j}_{11}-{j}_{22})+({j}_{12}-{j}_{21})\\ \,-({j}_{11}-{j}_{22})-({j}_{12}-{j}_{21}) & ({j}_{11}+{j}_{22})-({j}_{12}+{j}_{21})\end{array})\end{array}$$

7 $$\begin{array}{ll}{d}_{11}={D}_{T}(45^\circ )+{D}_{R}(45^\circ ) & {d}_{12}={D}_{R}(135^\circ )-{D}_{T}(135^\circ )\\ {d}_{21}={D}_{T}(45^\circ )-{D}_{R}(45^\circ ) & {d}_{22}={D}_{T}(135^\circ )+{D}_{R}(135^\circ )\end{array}$$

by combining the two sets of equation we get the equivalent Jones elements for this other orientation of the sample:

8 $$\begin{array}{rcl}{j}_{11} & = & (\,+\,{d}_{11}+{d}_{12}+{d}_{21}+{d}_{22})/2={D}_{T}(45^\circ )+{D}_{R}(135^\circ )\\ {j}_{12} & = & (\,-\,{d}_{11}+{d}_{12}-{d}_{21}+{d}_{22})/2={D}_{T}(135^\circ )-{D}_{T}(45^\circ )\\ {j}_{21} & = & (\,-\,{d}_{11}-{d}_{12}+{d}_{21}+{d}_{22})/2={D}_{R}(135^\circ )-{D}_{R}(45^\circ )\\ {j}_{22} & = & (\,+\,{d}_{11}-{d}_{12}-{d}_{21}+{d}_{22})/2={D}_{R}(45^\circ )+{D}_{T}(135^\circ )\end{array}$$

These coefficients are extracted using the sets of data recorded in the sample mount reference at angles −5° and 85° and added to Fig. 7 as the red dots (on the left block of four plots for the jones amplitude elements), showing consistency with the previously calculated black points.

Due to symmetry it must be possible to apply the same last set of equations to the previous measurements (0/90) and obtain a consistent result with the tilted set (blue points). This was done and added as the green set of points.

The legend in the top left plot of Fig. 7 allows interpretation of the curves describing the jones matrix element amplitudes. The two angles *M*°/*N*° represent the measurements performed at the angles in the birefringent plate reference. The arrow identifies if the jones matrix recovered is that in the reference frame parallel to the *xy* reference (that of the optical bench) or that of a reference frame rotated by 45°.

### Comparison with literature data

Validation of measurements can be seen in Fig. 7 as the over-plotted lines on the data points in the Jones matrix element amplitude, as well as the linear overlap of coloured blue lines on the diagonal phase plots are consistent with the identity Jones matrix

9 $${J}_{birefringent}=(\begin{array}{cc}{\tau }_{e}{e}^{-i{\varphi }_{e}} & 0\\ 0 & {\tau }_{o}{e}^{-i{\varphi }_{o}}\end{array})$$

with the amplitudes and phases given by:

10 $${\tau }_{e,o}=1-{\rho }_{e,o}^{2};\,{\rho }_{e,o}=\frac{1-{n}_{e,o}}{1+{n}_{e,o}};\,{\varphi }_{e,o}=2\pi t{n}_{e,o}\frac{{\nu }_{e,o}}{c}$$

The values used for the flat lines plotted in the figure are not a best fit of the data, but values for the ordinary and extraordinary sapphire axis at the peak of the THz emission (≈700 *GHz*) quoted in^35^ of ${n}_{o}=3.06\pm 0.02$ and ${n}_{e}=3.40\pm 0.02$ also consistent (within uncertainties) with Loewenstein’73^36^, Grischkowsky *et al*.^37^ and Kim *et al*.^38^. The red line in the bottom right plot is the overlay on the recovered difference in phase of the two ports with the differential phase in equation 10. The plots and model data are in good agreement. Small deviations of the recovered Jones matrix values could be due to the angle position uncertainty of the measurements.

### Collimated beam mapping

The measured sample is placed in a collimated beam ahead of the polarizer. To verify that the portions of the beam which are detected in the two ports are consistent and match at the sample (to avoid measuring with spatially uncorrelated beams) we acquired reference scans (no sample) with a small iris aperture (5 mm). The aperture was placed at the sample position. Its size small enough to allow coarse mapping of the beam but still large enough to avoid excessive diffraction losses. Acquiring a TDS scan for each position of the aperture raster-scan “map” of the collimated beam we identify the portions of the beam (in port I) which contribute to the modulation of each of the laser probe beams in ports T and R.

**Figure 8:**
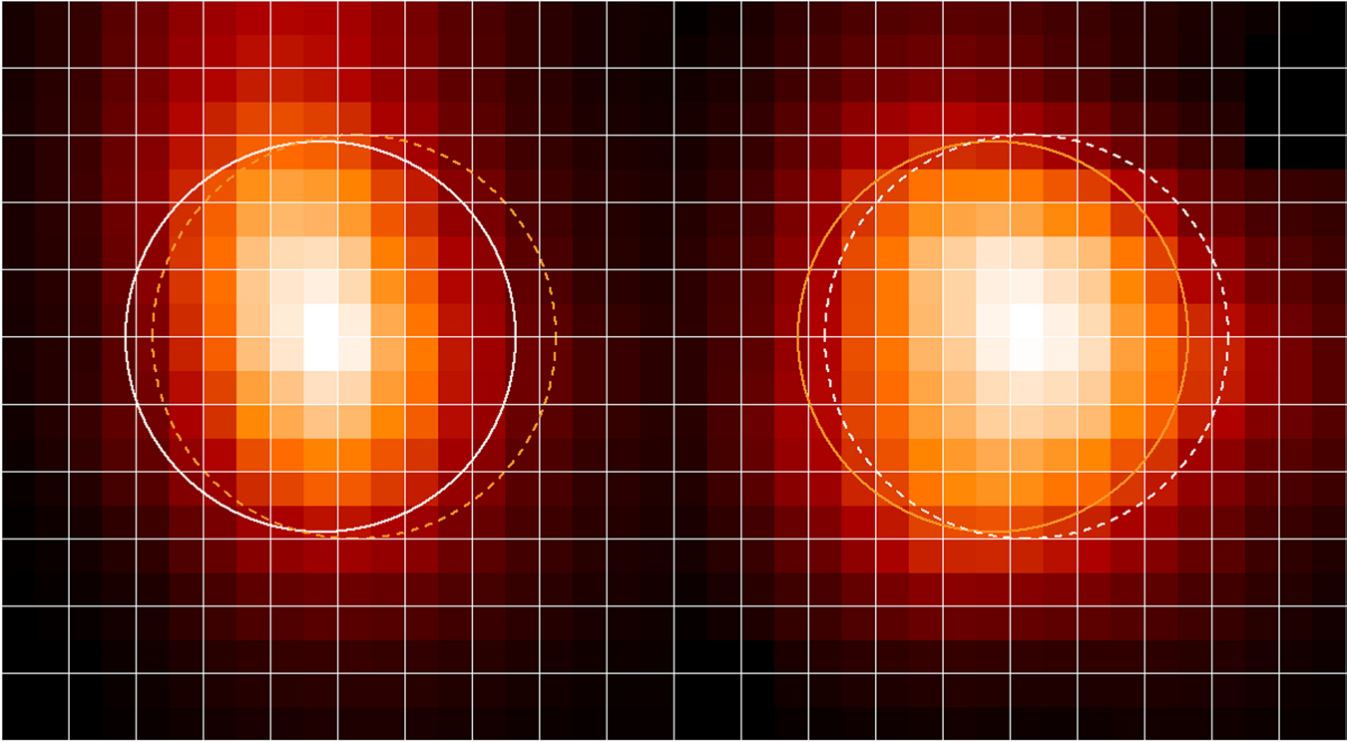
Amplitude maps of IG peak recorded in raster-scan mode. The rings show the cylinder envelope at which one sigma levels of power are contained (white in the beam and orange in the other for comparison).

The aperture was moved with an x-y stage across the available 5 cm beam and a very short time-domain interferogram recorded as the scan passed the main peak. The two images in Fig. 8 show the recorded amplitude at each of the positions of the aperture to produce a map of the beam at the intended position of the sample. The beam maps are sufficiently overlapped for the requirement of sample homogeneity to be satisfied.

## Discussion

We have designed, built and tested a dual-detector TDS system designed to allow polarimetry testing of homogeneous samples in collimated beams to minimize Gouy phase errors. The selection of the polarization in the THz part of the experiment allows to recover Jones matrix parameters for the sample without changing the operational angles of detection in the ZnTe crystal. To demonstrate this, we performed multiple measurements of a birefringent plate without prior knowledge of the axes orientation and recovered the Jones matrix consistent with actual axis position and with known literature data.

We have shown that while the recovered Jones matrix of a sample depends naturally on the sample angle with respect to the reference frame adopted, the recovery method of this does not, and can either be obtained when aligned, or recovered from any orthogonal angle pair and rotated consistently with its Jones matrix.

This system is thus useful for further studies of optical activity, polarization converters, circular dichroism and other properties of polarizing materials, which will alter the state of polarization from the input thus introducing degeneracies in parameter recovery from a standard TDS system.

Further improvement and implementations of this system can be obtained by investigating the use of additional refocusing mirrors to allow small sample measurements.

## Methods

The photo-conductive antenna was manufactured in house and is based on a parallel line-H architecture in Gold with 1 mm gap evaporated on a GaAs substrate. The PCA is biased at +70/−70 Volt with a 4.8 kHz frequency to which the lock-in amplifiers of the two detector ports are referenced. Details of the polarizer are provided in the article with the first polarizer available at QMC Instruments^32^ and the second obtained from HuiRuiOptics^33^. The sapphire plate used for the measurements (detailed in the Results section) was purchased from Crystran^39^ with C-axis parallel to disc (tolerance of =/−0.5 degrees and 5 *μm* tolerance on the thickness.

## Data Availability

The data contained in this paper is made available at https://tinyurl.com/y8l9ge9e.
